# Lymphocyte to Monocyte Ratio Predicts Resectability and Early Recurrence of Bismuth-Corlette Type IV Hilar Cholangiocarcinoma

**DOI:** 10.1007/s11605-018-04086-9

**Published:** 2019-01-22

**Authors:** Dingzhong Peng, Jiong Lu, Haijie Hu, Bei Li, Xiwen Ye, Nansheng Cheng

**Affiliations:** Department of Biliary Surgery, West China Hospital of Sichuan University, Chengdu, 614100 Sichuan Province China

**Keywords:** Lymphocyte to monocyte ratio, Resectability, Early recurrence, Bismuth-Corlette classification, Hilar cholangiocarcinoma

## Abstract

**Background:**

The objective of our research was to investigate the value of the lymphocyte to monocyte ratio (LMR) and its dynamic changes (LMRc) in predicting tumor resectability and early recurrence of radiologically resectable type IV hilar cholangiocarcinoma (HC).

**Methods:**

A total of 411 patients with radiologically resectable type IV HC were included. Data on their clinicopathologic characteristics, perioperative features, and survival outcomes were analyzed. Receiver operating characteristic (ROC) analysis was conducted to assess the ability of preoperative LMR (pre-LMR) to predict tumor resectability, and the ability of postoperative LMR (post-LMR) to discriminate between early and late recurrence. Survival curves were calculated using the Kaplan–Meier estimate. Univariate and multivariate logistic regression models were used to identify factors associated with resectability and early recurrence.

**Results:**

Of 411 patients with potentially curative type IV HC, 254 underwent curative surgery. The optimal cutoff value of pre-LMR as an indicator of resectability was 3.67, and the optimal cutoff value of post-LMR for detecting early recurrence was 4.10. In the multivariate logistic regression model, CA19-9 > 200 U/mL, pre-LMR ≤ 3.67, and tumor size > 3 cm were found to be independent risk factors for poor resectability. Moreover, multivariate analysis showed that LMRc, resection margin, AJCC N stage, and lymphovascular invasion were independent risk factors associated with early recurrence.

**Discussion:**

Pre-LMR is a valuable indicator of resectability and LMRc is a valuable predictor of early recurrence in patients with curative type IV HC.

## Introduction

Hilar cholangiocarcinoma (HC) is a lesion that arises from the biliary confluence and has a strong tendency to infiltrate into adjacent structures, such as the liver parenchyma, hepatic artery, portal vein, peripheral nerve system, and the bile duct.^1^ HC of Bismuth-Corlette type IV is a neoplasm that infiltrates the second-order biliary radicals of the bilateral hepatic ducts. To achieve negative resection margins for type IV HC, radical resection involving extended hepatectomy, caudate lobe resection, lymphadenectomy, vascular resection and reconstruction, and even pancreatoduodenectomy is advocated by numerous surgeons.^2, 3^ However, radicle resection for type IV HC is considered to be particularly challenging on account of the complexity of intact resection of locally advanced tumors.^4^ Therefore, the resectability rate is poor and the postoperative recurrence is high, as a result of which the survival outcome for HC is unsatisfactory, with the 5-year overall survival rate of 11–40%.^5–7^ To date, preoperative evaluation of the advancement and resectability of HC is mainly dependent on radiological examination.^8^ However, despite the progress of imaging diagnosis, radiologically resectable tumors are often later identified as being unsuitable for resection based on intraoperative discovery of extensive infiltration or distant metastasis. Therefore, from the perspective of selecting appropriate treatment strategies, it would be useful to identify biomarkers associated with the resectability and postoperative recurrence and survival rates of HC.

Recently, an increasing amount of research has been focusing on the effects of systemic inflammatory response (SIR) on oncogenesis, and such research has revealed that there is a significant relationship between SIR and poor tumor-specific survival in numerous cancers.^9–11^ In particular, the neutrophil to lymphocyte ratio (NLR), which is considered as a biomarker of SIR, has emerged as an independent prognostic factor for biliary tract cancer in operable patients and in advanced disease patients undergoing adjuvant therapy.^12, 13^ The lymphocyte to monocyte ratio (LMR) is another biomarker of SIR, with a combination of lymphocyte count and monocyte count. The LMR as a prognostic factor for patients with colorectal cancer and hepatocellular carcinoma was recently investigated in a number of studies.^14–16^ However, the value of LMR in predicting the resectability and early recurrence of type IV HC has never been investigated. Therefore, the current study was conducted to determine whether LMR could be used to predict the resectability and early recurrence rates of radiologically resectable type IV HC.

## Materials and Methods

### Patient Selection

A total of consecutive 411 patients who underwent surgery for radiologically resectable type IV HC at West China Hospital of Sichuan University between 2001 and 2012 were enrolled in this study. Patients with intrahepatic bile duct carcinoma or gallbladder carcinoma infringing the hilum, or radiologically unresectable malignancy, patients who had underwent preoperative chemotherapy and radiotherapy, and patients who died within 90 days of surgery were excluded. The Ethics Committee of West China Hospital of Sichuan University approved of this retrospective study and waived the need for informed consent.

### Preoperative Workup

Data from the patients’ medical history, physical examination, laboratory tests, and radiographic analyses (including contrast-enhanced ultrasound, contrast-enhanced computed tomography, and/or magnetic resonance cholangiography) were obtained. Preoperative biliary drainage was performed in patients with obstructive jaundice (total bilirubin > 85 μmol/L) by endoscopic retrograde cholangiopancreatography (ENBD) or percutaneous transhepatic cholangiodrainage (PTCD). Portal vein embolism (PVE) was performed 2–3 weeks before surgery for HC patients in whom the future remnant liver volume would be less than 40%.

### Treatment

Different surgical strategies were selected based on the preoperative and intraoperative evaluations of the HC patients, the strategies included extrahepatic bile duct resection and caudate lobectomy combined with hemihepatectomy or trisegmentectomy. Patients also underwent routine resection of the regional lymph nodes, including the hilar, pericholedochal, periportal, common hepatic artery, and peripancreatic lymph nodes. Palliative surgery, such as bypass operation and open biopsy, was performed in patients with the following intraoperative discoveries: (1) extensive bile duct infringement that precluded intact tumor resection, (2) invasion of major vascular system such as bilateral portal vein involvement that hampered vascular reconstruction, (3) unilateral hepatic lobe atropy combined with invasion of the contralateral portal vein or hepatic artery, and (4) evidence of distant metastases.^17^

Postoperative concurrent chemoradiotherapy or chemotherapy was recommended for patients with advanced TNM stage (III-IV) or microscopic/macroscopic positive resection margin. For 14 consecutive days every 28 days for 2 cycles, patients received a total radiation dose of 40 Gy delivered as a split course of 20 Gy in 10 fractions, followed by 375 mg/m^2^ of 5-FU or 1000 mg/m^2^ gemcitabine as maintenance chemotherapy.

### Data Collection

Details of patients’ demographics, clinical examination, laboratory tests, radiological analyses, surgical procedures, and survival outcomes were collected. Blood samples were collected 3 days before surgery for evaluation of preoperative LMR (pre-LMR) or preoperative NLR (pre-NLR), and within 4 months after surgery for postoperative LMR (post-LMR) or postoperative NLR (post-NLR). The largest dimension of tumor demonstrated on preoperative radiological examination was determined as the tumor size. Resected tumor samples were routinely sent to the Department of Pathology, where HC was confirmed by experienced pathologists. Tumor stage was determined using the tumor classification system published in the 8th edition of the American Joint Committee on Cancer (AJCC). Patients with R0 resection (microscopically tumor-free margins) and R1 resection (microscopically positive margins) were classified as resectable, while those with R2 resection (macroscopically positive margins) or palliative surgery were classified as unresectable. Postoperative complications were assessed with the Clavien-Dindo classification (CD).^18^ Those with more than one postoperative complication were considered to have the highest grade of severity.

### Follow-up Protocol

After discharge, all patients were routinely followed up every 3 months in the first year and every 6 months subsequently. Carbohydrate antigen 19-9 (CA19-9), carcinoembryonic antigen (CEA), and liver function tests and hepatic ultrasonography were performed for surveillance of recurrence. For those with suspected recurrence after curative resection, additional examinations, such as contrast-enhanced computed tomography and magnetic resonance imaging, were conducted for a definitive diagnosis. Patients with post-LMR lower than or equal to pre-LMR were confined to decreased LMRc group, while those with post-LMR higher than pre-LMR were confined to elevated LMRc group. The definition of NLRc was set accordingly. Disease-free survival (DFS) was defined as the interval between the date of surgery and the diagnosis of recurrence, or from the date of surgery to the date of last follow-up patients without recurrence. Overall survival (OS) was defined as the interval between the date of surgery and death of patients, or from the date of surgery to the date of last observation of surviving patients.

### Statistical Analysis

Data analysis was performed using the SPSS 19.0 software (SPSS Inc., Chicago, IL, USA). Comparisons between two groups were performed using the *t* test or Wilcoxon test for continuous variables and the chi-square test or Fisher’s exact test for categorical variables. Receiver operating characteristics (ROC) analysis was applied to the whole group and to the resectable subgroup separately to assess the ability of LMR to predict resectability and early recurrence, respectively. Survival was evaluated using Kaplan–Meier estimates, and differences in survival were analyzed by the log-rank test. To identify independent factors associated with resectability and early recurrence, variables were examined with univariate and multivariate logistic regression models. Two-tailed *p* values < 0.05 were considered statistically significant.

## Results

### Characteristics of the Study Population

The patient characteristics are shown in Table 1. A total of 411 type IV HC patients were considered to be suitable for resection based on preoperative radiological examinations. Among them, 254 patients (61.8%) underwent curative-intent surgery, while the remaining 157 patients (38.2%) were determined to have unresectable tumors as the intraoperative findings were indicative of aggressive tumor progression. The curative resection strategies included extrahepatic bile duct resection and caudate lobectomy combined with left hemihepatectomy (*n* = 132, 52.0%), right hemihepatectomy (*n* = 89, 35.0%), extended left hemihepatectomy (*n* = 8, 3.1%), extended right hemihepatectomy (*n* = 7, 2.8%), left trisegmentectomy (*n* = 12, 4.7%), and right trisegmentectomy (*n* = 6, 2.4%). The median of bile ducts reconstructed and hepaticojujunostomies performed was 2 (range, 1–3). Regional lymph node resection was routinely performed in all patients undergoing curative surgery. Of 370 patients with obstructive jaundice, 227 patients who had total bilirubin levels above 85 μmol/L underwent preoperative biliary drainage: 72 patients underwent ENBD and 155 patients underwent PTCD. PVE was performed in 24 patients.

**Table 1:** Characteristics of the whole study population

Variables	Overall (*n* = 411)	Resectable group (*n* = 254)	Unresectable group (*n* = 157)
Age (years)^1^	60 (26–82)	61 (26–82)	58 (28–81)
Gender			
Male	263	159	104
Female	147	94	53
BMI^2^	22.3 (17.2–28.6)	22.0 (17.2–28.6)	23.0 (17.6–28.6)
ASA score			
1	12 (2.9%)	6 (2.4%)	6 (3.8%)
2	224 (54.5%)	140 (5.5%)	84 (53.5%)
3	175 (42.6%)	108 (42.5%)	67 (42.7%)
Total bilirubin (μmol/L)^2^	224.74 ± 162.22	206.72 ± 172.04	253.90 ± 140.60
CA-199 (U/mL)^2^	450.19 ± 369.53	414.38 ± 369.55	419.30 ± 363.23
Albumin (g/L)^2^	37.05 ± 5.23	36.94 ± 5.18	37.19 ± 5.39
Preoperative neutrophil count (× 10^9^/L)^2^	4.77 ± 2.52	4.61 ± 2.41	5.03 ± 2.68
Preoperative monocyte count (× 10^9^/L)^2^	0.52 ± 0.19	0.52 ± 0.19	0.52 ± 0.19
Preoperative lymphocyte count (× 10^9^/L)^2^	2.05 ± 0.79	2.13 ± 0.81	1.91 ± 0.75
Postoperative neutrophil count (× 10^9^/L)^2^	4.40 ± 1.37	4.23 ± 1.22	4.68 ± 1.55
Postoperative monocyte count (× 10^9^/L)^2^	0.51 ± 0.17	0.50 ± 0.17	0.52 ± 0.17
Postoperative lymphocyte count (× 10^9^/L)^2^	2.29 ± 0.88	2.27 ± 0.76	2.34 ± 1.04
Total lymph nodes evaluated^1^	4 (1–12)	3 (1–9)	5 (1–12)
Positive lymph node number^1^	2 (0–10)	0 (0–7)	4 (1–10)
Operative time (min)^1^	240.00 (80–720)	250 (110–720)	180 (80–500)
Blood loss (mL)^2^	500 (50–2000)	600 (100–2000)	300 (50–1000)
Blood transfusion	123 (30.0%)	84 (33.0%)	39 (24.8%)
Any complication	184 (44.8%)	141 (55.5%)	43 (27.3%)
Major complication (Clavien-Dindo II-IV)	117 (28.5%)	93 (36.6%)	24 (15.3%)
Hospital stay (days)^1^	18 (5–113)	18 (5–113)	17 (5–43)
Preoperative hospital stay (days)^1^	7 (2–44)	7 (2–44)	7 (3–33)
Postoperative adjuvant therapy	154 (37.3%)	117 (46.1%)	37 (23.6%)

^1^Parameters are presented as median and range

^2^Parameters are presented as mean ± SD

The pre-LMR of patients who underwent PVE was higher than that of the patients who did not undergo PVE, but the difference was not statistically significant in the entire patient group (4.18 ± 0.96 vs 3.96 ± 0.96, *p* = 0.282) or in the resectable group (4.29 ± 0.85 vs 4.11 ± 0.77, *p* = 0.344). In total, 21 patients died within 90 days after surgery and were excluded from the analysis to reduce the impact of postoperative complications when assessing the recurrence rates and calculating the optimal cutoff point for early recurrence.^19, 20^ The postoperative complication rate after surgery was 44.8% (*n* = 184), which included 117 patients with CD II or higher complications. Major complications (CD II-III) consisted of bile leakage (*n* = 28), peritoneal cavity infection (*n* = 14), lung infection (*n* = 18), sepsis (*n* = 4), hemorrhage (*n* = 13), hepatic failure (*n* = 18), renal failure (*n* = 2), stress ulcer (*n* = 7), and others (*n* = 13). In the entire patient group, post-LMR of patients with Clavien-Dindo grade I complications was significantly higher than that of patients with major complications (6.55 ± 5.89 vs 4.68 ± 3.27, *p* = 0.006), as was in the resectable group (7.35 ± 6.47 vs 4.87 ± 3.54, *p* = 0.004). However, no significant association was detected between post-LMR and infectious complications neither in the entire patient group (4.59 ± 3.29 vs 4.80 ± 3.28, *p* = 0.738) nor in the resectable group (4.88 ± 3.62 vs 4.85 ± 3.48, *p* = 0.963).

### Survival

**Fig. 1:**
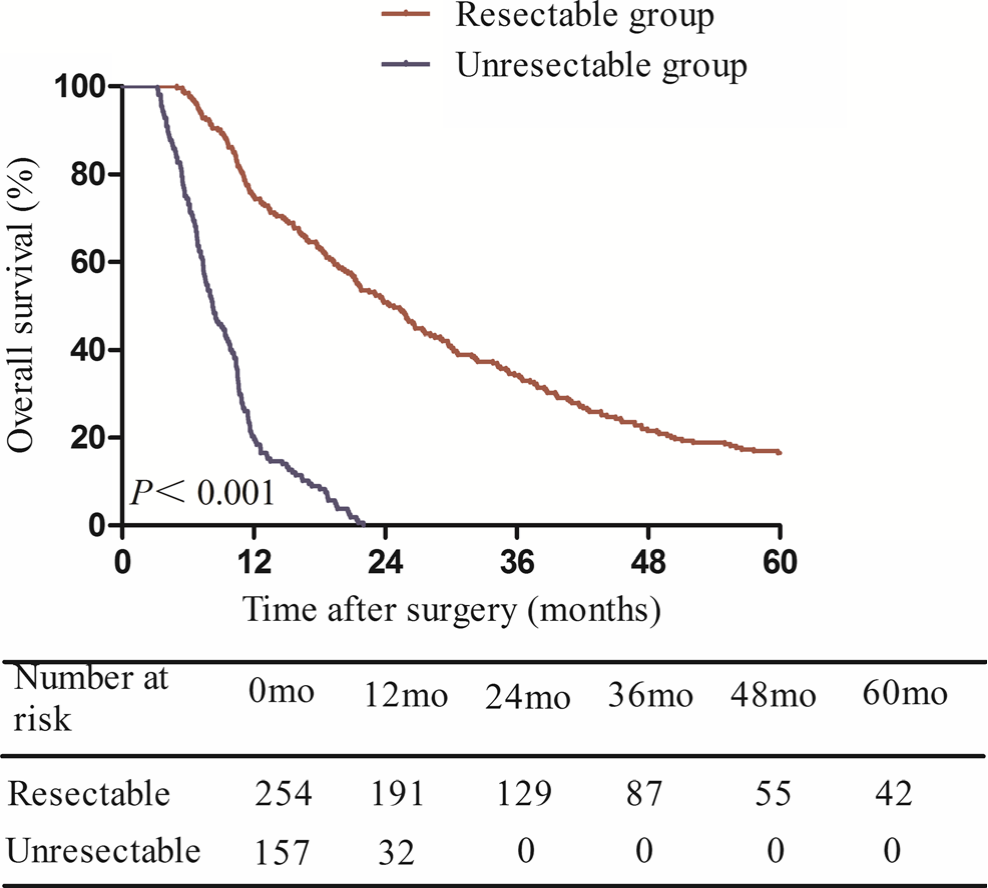
Kaplan–Meier analysis showing overall survival in patients with resectable and unresectable type IV HC

The median follow-up time for the entire patient group was 15 months (range, 4–115 months). In the resectable group, the median overall survival time was 24.6 months, and the 1-, 3-, 5-year overall survival rates were 75.2, 34.2, and 16.5%, respectively. In patients with unresectable tumors, the median overall survival time was 8.3 months, and the 1-, 3-, 5- year overall survival rates were 20.4, 0, and 0%, respectively (Fig. 1).

**Fig. 2:**
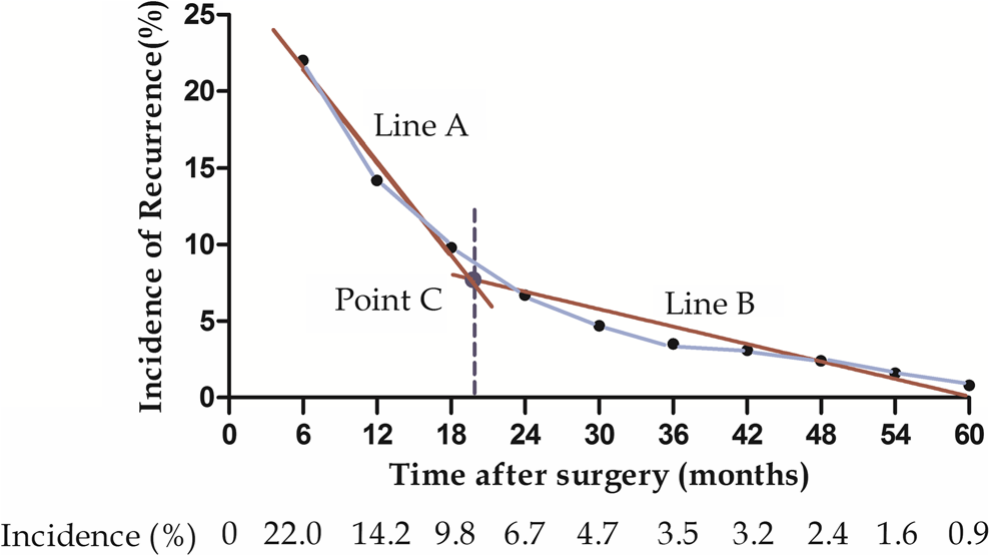
Recurrence rate after curative-intent surgery for type IV HC patients at 6-month interval

In the resectable group, overall 185 (72.8%) patients experienced tumor recurrence after curative resection. The 1-, 3-, and 5-year recurrence rates were 36.2, 61.0, and 68.9%, respectively. Decision of the optimal cutoff point for early and late recurrence of type IV HC was made based on the recurrence rate calculated at 6-month time points. Recurrence was divided into two periods from 6 to 60 months according to the linear regression analysis. The two straight lines were Line A (y = − 0.9559x + 26.676, R^2^ = 0.97) and Line B (y = − 0.1913x + 11.544, R^2^ = 0.90). The intercept point of the two lines was C point (19.7, 7.8). Therefore, 20 months was defined as the cutoff point to distinguish early from late recurrence for resectable type IV HC (Fig. 2).

**Fig. 3:**
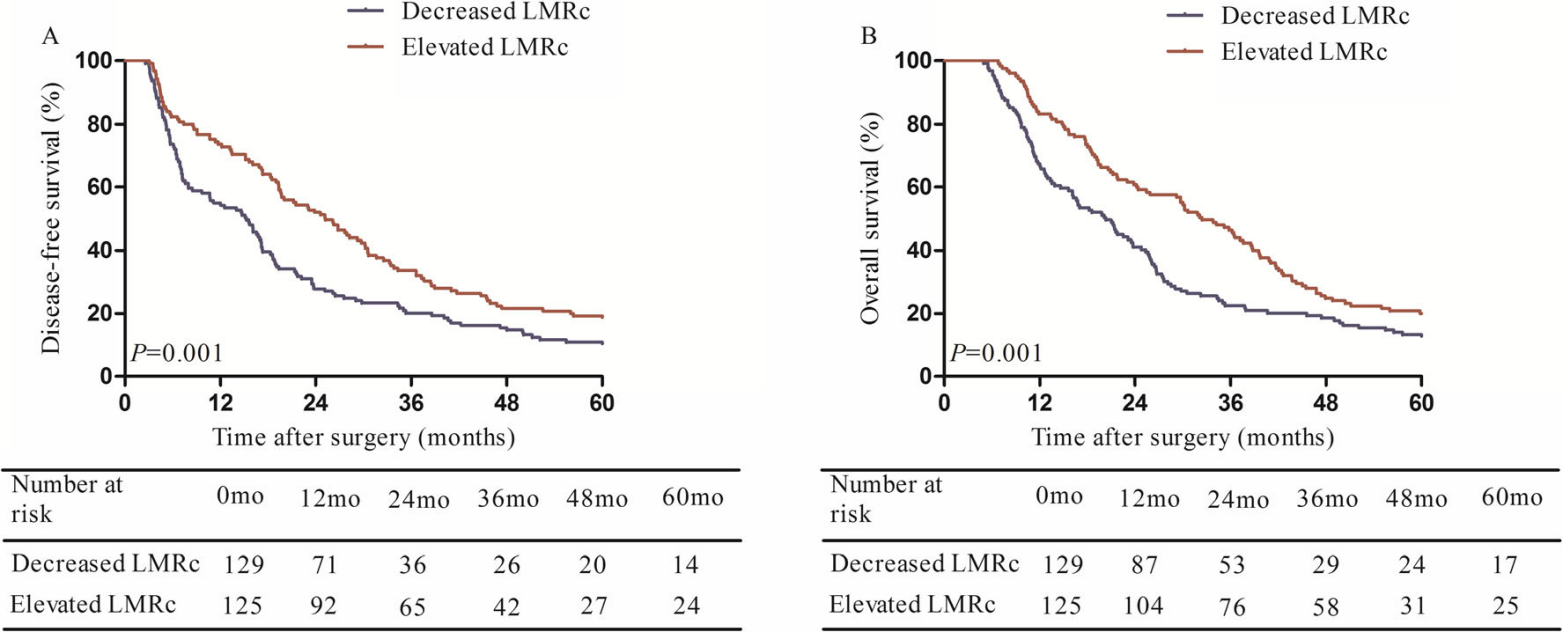
**a** The 5-year disease-free survival rate of the low LMR group was significantly lower than that of the high LMR group; **b** the 5-year overall survival rate of the high LMR group was significantly higher than that of the low LMR group

Patients with resectable type IV HC were stratified into two groups according to the dynamic changes in the LMR: the decreased LMRc group (*n* = 129) and the elevated LMRc group (*n* = 125). The 5-year disease-free survival rate of the decreased LMRc group was significantly lower than that of the elevated LMRc group (Fig. 3a), as was the 5-year overall survival rate (Fig. 3b).

### ROC Analysis

**Fig. 4:**
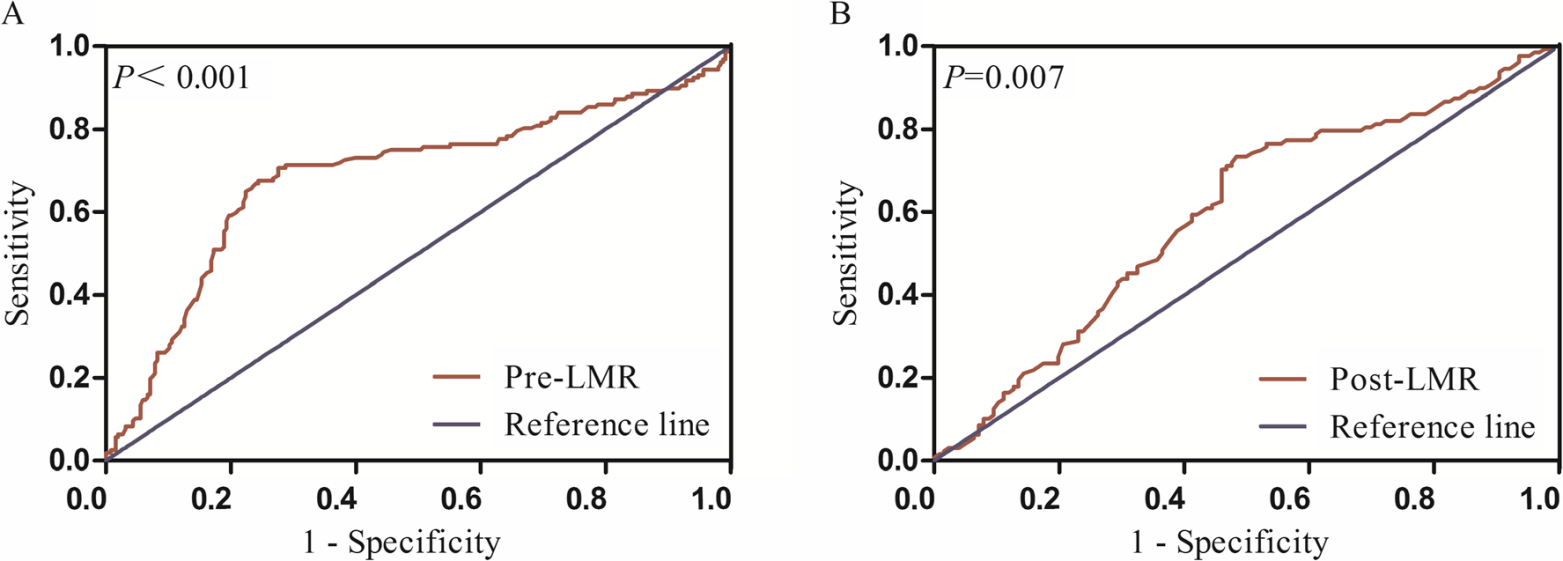
**a** ROC analysis of pre-LMR for determining the resectability for radiologically resectable type IV HC; **b** ROC analysis of post-LMR for determining early recurrence of resectable type IV HC after curative resection

ROC analysis of pre-LMR for detecting resectability is shown in (Fig. 4a). The pre-LMR value for discriminating resectable type IV HC tumors from unresectable type IV HC patients was shown to be significant (area under the ROC curve [AUC] = 0.687, 95% confidence interval [CI] 0.631–0.744, *p <* 0.001). ROC curve analysis was used to calculate the ideal pre-LMR cutoff value for the prediction of resectability; it was found to be 3.67, with a sensitivity of 76.4%, and specificity of 66.9%. Furthermore, ROC analysis was applied in the resectable group to verify the value of post-LMR as an indicator of early recurrence (Fig. 4b). The optimal cutoff point for post-LMR was 4.10, with a sensitivity of 73.4% and a specificity of 51.6%. AUC was 0.598, with a 95% CI of 0.527–0.668 (*p* = 0.007).

### Univariate and Multivariate Analysis of Clinicopathologic Indicators of Resectability and Early Recurrence

Table 2 demonstrates the correlation of the resectability rate with various preoperative clinicopathologic characteristics. Univariate analysis indicated that elevated CA19-9 levels, increased total bilirubin, high BMI, decreased pre-LMR, decreased pre-NLR, and bigger tumor size were negatively associated with the resectability of type IV HC. By contrast, gender, age, albumin, ASA score, and PVE were not useful predictors for resectability. Multivariate analysis using a logistic regression model demonstrated that CA19-9, pre-LMR, and tumor size were significant factors associated with the resectability of radiologically curative type IV HC.

**Table 2:** Analysis for preoperative variables associated with resectability of type IV HC patients

Variables	Univariate analysis			Multivariate analysis	
	Resectable group (*n* = 207)	Unresectable group (*n* = 81)	*p* value	HR (95% CI)	*p* value
Gender			0.486		
Male	159	104			
Female	94	53			
Age (years)			0.323		
**<** 70	215	127			
≥ 70	39	30			
CA-199 (U/mL)			*< 0.001*	1.970 (1.149–3.378)	*0.014*
≤ 200	106	37			
> 200	148	120			
Total bilirubin (μmol/L)	206.72 ± 172.05	253.90 ± 140.60	*0.004*	1.001 (0.999–1.002)	0.450
Albumin (g/L)			0.754		
≤ 40	188	114			
> 40	66	43			
BMI			*0.038*	1.584 (0.944–2.659)	0.082
≤ 21	100	46			
> 21	154	111			
ASA score			0.686		
1	6	6			
2	140	84			
3	108	67			
Pre-NLR			*0.041*	1.517 (0.900–2.556)	0.117
< 3	183	98			
≥ 3	71	59			
Pre-LMR			*< 0.001*	0.181 (0.111–0.296)	*< 0.001*
≤ 3.67	62	105			
> 3.67	192	52			
Tumor size (cm)			*< 0.001*	6.218 (3.705–10.435)	*< 0.001*
≤ 3	212	69			
> 3	42	88			
Portal vein embolization					
Yes	17	7	0.348		
No	237	150			

The Italic emphasis (originally boldfaced) in Tables 2 and 3 specified the variables that were statistically significant in the univariate and multivariate logistic regression analysis (two-tailed *P* values < 0.05)

A separate analysis was performed in the resectable group to identify predictors correlated with early recurrence (Table 3). In the univariate analysis, increased total bilirubin, CA19-9 > 200 U/mL, lower post-LMR, decreased LMRc, R1 resection, higher AJCC N stage, and positive lymphovascular invasion were associated with early recurrence. Multivariate analysis using the logistic regression model demonstrated that LMRc, R1 resection, AJCC N stage, and lymphovascular invasion were independent risk factors for early recurrence following curative-intent resection of type IV HC.

**Table 3:** Analysis of risk variables for early recurrence (≤20 months) of type IV HC after curative surgery

Variables	Univariate analysis			Multivariate analysis	
	Early recurrence group (*n* = 127)	20-month recurrence-free group (*n* = 126)	*p* value	HR (95% CI)	*p* value
Gender			0.407		
Male	83	76			
Female	44	50			
Age			0.203		
< 70	112	103			
≥ 70	16	23			
Total bilirubin (μmol/L)	229.85 ± 189.53	183.23 ± 149.37	*0.031*	1.001 (0.999–1.003)	0.170
CA-199 (U/mL)			*0.032*	1.750 (0.986–3.107)	0.057
< 200	45	61			
≥ 200	83	65			
Albumin (g/L)			0.941		
≤ 40	95	93			
> 40	33	33			
Pre-NLR			0.144		
< 3	87	96			
≥ 3	41	30			
Post-NLR			0.139		
< 3	103	110			
≥ 3	25	16			
NLRc			0.446		
Elevated	63	56			
Decreased	65	70			
Pre-LMR			0.421		
≤ 3.67	34	28			
> 3.67	94	98			
Post-LMR			*< 0.001*	0.591 (0.266–1.312)	0.196
≤ 4.10	94	61			
> 4.10	34	65			
LMRc			*< 0.001*	0.421 (0.188–0.942)	*0.035*
Elevated	48	77			
Decreased	80	49			
Resection margin			*0.002*	2.133 (1.112–4.091)	*0.023*
R0	75	97			
R1	53	29			
Tumor differentiation			0.065		
Well	25	37			
Moderate	85	80			
Poor	18	9			
AJCC T stage			0.151		
T1	5	12			
T2	77	66			
T3	37	43			
T4	9	5			
AJCC N stage			*0.001*	2.061 (1.344–3.162)	*0.001*
N0	63	92			
N1	45	24			
N2	20	10			
AJCC stage			0.094		
I	4	8			
II	40	51			
III	64	57			
IV	20	10			
Portal vein invasion			0.132		
Positive	28	38			
Negative	100	88			
Hepatic artery invasion			0.119		
Positive	18	10			
Negative	110	116			
Lymphovascular invasion			*< 0.001*	2.744 (1.345–5.595)	*0.006*
Positive	41	15			
Negative	87	111			
Perineural invasion			0.383		
Positive	86	91			
Negative	42	35			
Tumor size (cm)			*0.021*	1.794 (0.826–3.897)	0.140
≤ 3	100	112			
> 3	28	14			
Adjuvant therapy			0.212		
Yes	54	63			
No	74	63			

The Italic emphasis (originally boldfaced) in Tables 2 and 3 specified the variables that were statistically significant in the univariate and multivariate logistic regression analysis (two-tailed *P* values < 0.05)

## Discussion

Radical resection remains the only treatment option for HC patients to achieve long-term survival (Fig. 1), with resectability rate ranging from 32 to 80%.^21–23^ Considering the difficulty of curative-intent surgery for type IV HC, it was bracing to find that the radical resection rate is 61.8% in our institution. This encouraging result probably attributed to relatively strict criteria of resectability based on preoperative images: patients with tumor infiltration along the bile duct beyond both right side of the umbilicus of the left portal vein and the cranio-ventral side of the right portal vein or its anterior branch, and patients with unreconstructable vascular invasion with end-to-end anastomosis were deemed as unresectable and excluded.^24^ However, our criteria of resectability and operation may not be applicable in all institutions, which is a limitation of current research.

To our knowledge, no other report has ever revealed the significance of pre-LMR in predicting the resectability for type IV HC patients. Our study has for the first time indicated that pre-LMR was a useful predictor of resectability with an AUC of 0.687, at an optimal cutoff value of 3.67 (Fig. 4a). With increasing pre-LMR level, the resection rates raise from 37.1% in patients with pre-LMR ≤ 3.67 to 78.7% in those with pre-LMR > 3.67 (*p* < 0.001). Multivariate model has further verified pre-LMR ≤ 3.67 to be an unfavorable predictor of resectability. Previous research has revealed that pre-LMR is adversely correlated with tumor-related factors such as CA19-9, AJCC N stage, and distant metastasis.^25, 26^ This inverse association of low pre-LMR with indicators of advanced disease stage may explain why low pre-LMR was associated with a low resection rate. PVE has been reported to significantly improve resection outcomes.^27^ In our cohort, the impact of PVE on pre-LMR and tumor resectability was found to be insignificant, this difference is probably attributed to a statistical bias caused by the limited sample size of patients with PVE in this study. Thus, despite this finding, pre-LMR could still act as a complementary tool for the preoperative assessment of resectability in patients with radiographically curative type IV HC.

CA19-9 is the most investigated tumor biomarker and is considered as a predictor of resectability in numerous cancers.^28, 29^ In accordance with previous studies, the resectability rate of patients with CA19-9 > 200 U/mL was significantly lower than those with CA19-9 ≤ 200 U/mL (55.2 vs 74.1%, *p <* 0.001). However, two variables that might affect CA19-9 level were not taken into account in our study: First, previous studies excluded Lewis negative population due to their incapability to secrete CA19-9;^30^ second, former researches considered the impact of obstructive jaundice and cholangitis on CA19-9 by stratifying cohort according to hyperbilirubinemia.^31^ The present study neither excluded the Lewis negative population nor stratified patients based on bilirubin level. This might be a serious limitation of our study.

Tumor size > 3 cm is considered as a major risk factor for unresectability of HC.^32^ Hu et al.^28^ demonstrated that HC patients with tumor size > 3 cm were more likely to have unresectable tumors. Consistently, the resectability rate of type IV HC with tumor size > 3 cm was significantly lower than those with tumor size ≤ 3 cm in our cohort (43.0 vs 82.8%, *p* < 0.001). As the diameter of unresectable tumors could not be determined and measuring the size of only the resected tumors would introduce a bias, in our study, tumor size was determined on the basis of preoperative radiologic examination. According to the abovementioned results, we believe that patients with potentially curative type IV HC who have an pre-LMR ≤ 3.67, CA19-9 ≤ 200 U/mL, and radiologically diagnosed tumor size > 3 cm may be unsuitable for resection and should therefore be further evaluated with diagnostic laparoscopy.

Early recurrence after curative surgery has been associated with poor prognosis in hepatic carcinoma.^19, 33, 34^ Zhang et al.^19^ defined radiologically diagnosed recurrence within 2.5 years after curative surgery as early recurrence in HC patients. The difficulty to achieve negative resection margin for type IV HC renders relatively worse prognosis.^35^ For the present cohort, we determined that 20 months was the optimal cutoff point to define early recurrence for type IV HC after curative resection (Fig. 2). We also tried to identify the factors associated with early, as these could be useful for guiding surveillance, adjuvant therapy, and follow-up in patients who are likely to have early recurrence.

The prognostic value of post-LMR and LMRc for type IV HC has never been reported in previous studies. ROC analysis indicated that post-LMR was not a useful predictor of early recurrence, with an AUC less than 0.60 (Fig. 4b). Multivariate analysis also demonstrated that LMRc, rather than pre-LMR or post-LMR, was a predictor of early recurrence. Decreased LMRc reflects relatively reduced lymphocyte counts or increased monocyte counts. Low lymphocyte count is known to be an indicator of weakened T lymphocyte-mediated antitumor ability.^36^ That is, the host immune response against tumor progression and metastasis is suppressed due to lymphocytopenia.^37^ On the other hand, circulating monocytes are recruited to the tumor microenvironment, where they subsequently promotes tumor progression.^38^ Moreover, monocytes within the tumor tissue differentiate into macrophages, which are capable of immunosuppression, metastasis promotion, and angiogenesis.^39^ In summary, a decreased LMRc in type IV HC patients after surgery reflects two aspects of tumor progression—the advancement of malignancy and suppression of the immune system, which explains why patients with decreased LMRc were more likely to develop early recurrence in our research. We also noted that decreased LMRc was associated with shorter disease-free survival and overall survival. With regard to the impact of postoperative complications on post-LMR and consequently LMRc, the results demonstrated that, while patients with severe postoperative complication had lower post-LMR, those with infectious complication did not. Decreased post-LMR could be the result of lymphopenia triggered by complications such as hemorrhage and liver failure. It is possible that major postoperative complications impair cell-mediated immunity, which results in an early recurrence.

Other factors strongly associated with early recurrence in our cohort included AJCC N stage, lymphovascular invasion, and resection margin. In keeping with our results, a positive relationship has been shown between N stage and early recurrence for potentially resectable HC.^19^ While the pathologic classification of previous studies was mainly based on AJCC 7th edition, in which lymph node metastases were stratified according to the distribution of lymph nodes, our cohort followed the 8th edition of AJCC staging, which uses the number of positive lymph nodes for classification. Similar to our results, lymphovascular invasion has been strongly associated with poor oncological prognosis in type IV HC patients.^35^ Further, surgery-related factors such as the resection margin have also been shown to influence oncologic outcomes,^40^ as patients with an R0 margin had a decreased risk of early recurrence in our study. This finding further stresses on the importance of R0 resection to prevent early recurrence and that, to guarantee R0 resection, measurements such as intraoperative frozen section examination should be routinely performed if possible.^41^

Several limitations of the current study should be considered. This was a retrospective and uncontrolled study that was conducted at a single center, which means that a selection bias was inevitable. Moreover, both monocyte counts and lymphocyte counts could have been affected by factors such as medications or postoperative complications, which were not sufficiently taken into account. Finally, our criteria of resectability and operation may not be shared by all centers. Despite these limitations, nomograms will be constructed on the basis of present research to predict resectability and early recurrence in patients with type IV HC in the future.

## Conclusion

LMR is superior to NLR in predicting resectability and early recurrence for type IV HC. According to the above findings, decreased pre-LMR is associated with a poor resectability rate and may help identify patients who require further evaluation with diagnostic laparoscopy. Furthermore, patients with decreased LMRc are more likely to develop early recurrence and should be closely followed up. Still, it should be noted that it is not realistic to estimate resectability or early recurrence on the basis of a single biomarker. LMR estimation will be useful only if it is used along with comprehensive surveillances such as radiologic examinations and biological tests.

## References

[1] Gerhards MF, van Gulik TM, Bosma A, ten Hoopen-Neumann H, Verbeek PCM, Gonzalez DG (1999). Long-term survival after resection of proximal bile duct carcinoma (Klatskin tumors). World Journal of Surgery.

[2] Weiss MJ, Cosgrove D, Herman JM, Rastegar N, Kamel I, Pawlik TM (2014). Multimodal treatment strategies for advanced hilar cholangiocarcinoma. Langenbeck Arch Surg..

[3] Baton O, Azoulay D, Adam DV, Castaing D (2007). Major hepatectomy for hilar cholangiocarcinoma type 3 and 4: prognostic factors and longterm outcomes. J Am Coll Surg..

[4] Govil S, Reddy MS, Rela M (2014). Surgical resection techniques for locally advanced hilar cholangiocarcinoma. Langenbeck’s archives of surgery..

[5] Jarnagin WR, Fong Y, DeMatteo RP, Gonen M, Burke EC, Bodniewicz J (2001). Staging, resectability, and outcome in 225 patients with hilar cholangiocarcinoma. Annals of Surgery.

[6] Kobayashi A, Miwa S, Nakata T, Miyagawa S (2010). Disease recurrence patterns after R0 resection of hilar cholangiocarcinoma. Brit J Surg.

[7] Molina V, Sampson J, Ferrer J, Diaz A, Ayuso JR, Sanchez-Cabus S (2017). Surgical treatment of perihilar cholangiocarcinoma: early results of en bloc portal vein resection. Langenbeck Arch Surg..

[8] Soares KC, Kamel I, Cosgrove DP, Herman JM, Pawlik TM (2014). Hilar cholangiocarcinoma: diagnosis, treatment options, and management. Hepatobiliary Surgery and Nutrition..

[9] McMillan DC, Canna K, McArdle CS (2003). Systemic inflammatory response predicts survival following curative resection of colorectal cancer. Brit J Surg..

[10] Zhang Weiwei, Liu Kejun, Ye Bin, Liang Weijiang, Ren Yazhou (2017). Pretreatment C-reactive protein/albumin ratio is associated with poor survival in patients with stage IB-IIA cervical cancer. Cancer Medicine.

[11] Pang Q, Zhang LQ, Wang RT, Bi JB, Zhang JY, Qu K (2015). Platelet to lymphocyte ratio as a novel prognostic tool for gallbladder carcinoma. World Journal of Gastroenterology..

[12] McNamara MG, Templeton AJ, Maganti M, Walter T, Horgan AM, McKeever L (2014). Neutrophil/lymphocyte ratio as a prognostic factor in biliary tract cancer. European Journal of Cancer..

[13] Grenader T, Nash S, Plotkin Y, Furuse J, Mizuno N, Okusaka T (2015). Derived neutrophil lymphocyte ratio may predict benefit from cisplatin in the advanced biliary cancer: the ABC-02 and BT-22 studies. Annals of Oncology: Official Journal of the European Society for Medical Oncology..

[14] Shen SL, Fu SJ, Huang XQ, Chen B, Kuang M, Li SQ (2014). Elevated preoperative peripheral blood monocyte count predicts poor prognosis for hepatocellular carcinoma after curative resection. BMC Cancer..

[15] Chan JCY, Chan DL, Diakos CI, Engel A, Pavlakis N, Gill A (2017). The Lymphocyte-to-Monocyte Ratio is a Superior Predictor of Overall Survival in Comparison to Established Biomarkers of Resectable Colorectal Cancer. Annals of Surgery..

[16] Ozawa T, Ishihara S, Kawai K, Kazama S, Yamaguchi H, Sunami E (2015). Impact of a lymphocyte to monocyte ratio in stage IV colorectal cancer. J Surg Res..

[17] Dumitrascu T, Chirita D, Ionescu M, Popescu I (2013). Resection for Hilar Cholangiocarcinoma: Analysis of Prognostic Factors and the Impact of Systemic Inflammation on Long-term Outcome. Journal of Gastrointestinal Surgery..

[18] Dindo D, Demartines N, Clavien PA (2004). Classification of surgical complications—A new proposal with evaluation in a cohort of 6336 patients and results of a survey. Annals of Surgery..

[19] Zhang XF, Beal EW, Chakedis J, Chen Q, Lv Y, Ethun CG (2018). Defining Early Recurrence of Hilar Cholangiocarcinoma After Curative-intent Surgery: A Multi-institutional Study from the US Extrahepatic Biliary Malignancy Consortium. World Journal of Surgery..

[20] Groot Koerkamp B, Wiggers JK, Allen PJ, Besselink MG, Blumgart LH, Busch OR (2015). Recurrence Rate and Pattern of Perihilar Cholangiocarcinoma after Curative Intent Resection. J Am Coll Surg..

[21] Nagino M (2012). Perihilar cholangiocarcinoma: a surgeon’s viewpoint on current topics. Journal of Gastroenterology..

[22] Valero V, Cosgrove D, Herman JM, Pawlik TM (2012). Management of perihilar cholangiocarcinoma in the era of multimodal therapy. Expert Review of Gastroenterology & Hepatology..

[23] Cameron JL, Pitt HA, Zinner MJ, Kaufman SL, Coleman J (1990). Management of proximal cholangiocarcinomas by surgical resection and radiotherapy. Am J Surg..

[24] Ji GW, Zhu FP, Wang K, Jiao CY, Shao ZC, Li XC (2017). Clinical Implications of Biliary Confluence Pattern for Bismuth-Corlette Type IV Hilar Cholangiocarcinoma Applied to Hemihepatectomy. Journal of Gastrointestinal Surgery: Official Journal of the Society for Surgery of the Alimentary Tract..

[25] Lin JP, Lin JX, Cao LL, Zheng CH, Li P, Xie JW (2017). Preoperative lymphocyte-to-monocyte ratio as a strong predictor of survival and recurrence for gastric cancer after radical-intent surgery. Oncotarget.

[26] Hu RJ, Ma JY, Hu G (2018). Lymphocyte-to-monocyte ratio in pancreatic cancer: Prognostic significance and meta-analysis. Clinica Chimica Acta; International Journal of Clinical Chemistry.

[27] Seyama Y, Kubota K, Sano K, Noie T, Takayama T, Kosuge T (2003). Long-term outcome of extended hemihepatectomy for hilar bile duct cancer with no mortality and high survival rate. Annals of Surgery..

[28] Hu HJ, Mao H, Tan YQ, Shrestha A, Ma WJ, Yang Q (2016). Clinical value of preoperative serum CA 19-9 and CA 125 levels in predicting the resectability of hilar cholangiocarcinoma. SpringerPlus..

[29] Hartwig W, Strobel O, Hinz U, Fritz S, Hackert T, Roth C (2013). CA19-9 in potentially resectable pancreatic cancer: perspective to adjust surgical and perioperative therapy. Annals of Surgical Oncology..

[30] Chen T, Zhang MG, Xu HX, Wang WQ, Liu L, Yu XJ (2015). Preoperative Serum CA125 Levels Predict the Prognosis in Hyperbilirubinemia Patients With Resectable Pancreatic Ductal Adenocarcinoma. Medicine.

[31] Rerknimitr R, Angsuwatcharakon P, Ratanachu-ek T, Khor CJL, Ponnudurai R, Moon JH (2013). AsiaPacific consensus recommendations for endoscopic and interventional management of hilar cholangiocarcinoma. Journal of Gastroenterology and Hepatology..

[32] Murad SD, Kim WR, Harnois DM, Douglas DD, Burton J, Kulik LM (2012). Efficacy of Neoadjuvant Chemoradiation, Followed by Liver Transplantation, for Perihilar Cholangiocarcinoma at 12 US Centers. Gastroenterology..

[33] Portolani N, Coniglio A, Ghidoni S, Giovanelli M, Benetti A, Tiberio GAM (2006). Early and late recurrence after liver resection for hepatocellular carcinoma—Prognostic and therapeutic implications. Annals of Surgery..

[34] Zhang XF, Beal EW, Bagante F, Chakedis J, Weiss M, Popescu I (2018). Early versus late recurrence of intrahepatic cholangiocarcinoma after resection with curative intent. Brit J Surg..

[35] Li B, Xiong XZ, Zhou Y, Wu SJ, You Z, Lu J (2017). Prognostic value of lymphovascular invasion in Bismuth-Corlette type IV hilar cholangiocarcinoma. World Journal of Gastroenterology..

[36] Menges T, Engel J, Welters I, Wagner RM, Little S, Ruwoldt R (1999). Changes in blood lymphocyte populations after multiple trauma: Association with posttraumatic complications. Crit Care Med.

[37] Iwase R, Shiba H, Haruki K, Fujiwara Y, Furukawa K, Futagawa Y (2013). Post-operative lymphocyte count may predict the outcome of radical resection for gallbladder carcinoma. Anticancer Research..

[38] Ying HQ, Deng QW, He BS, Pan YQ, Wang F, Sun HL (2014). The prognostic value of preoperative NLR, d-NLR, PLR and LMR for predicting clinical outcome in surgical colorectal cancer patients. Medical Oncology..

[39] Chanmee T, Ontong P, Konno K, Itano N (2014). Tumor-associated macrophages as major players in the tumor microenvironment. Cancers..

[40] Zhang XF, Squires MH, Bagante F, Ethun CG, Salem A, Weber SM (2018). The Impact of Intraoperative Re-Resection of a Positive Bile Duct Margin on Clinical Outcomes for Hilar Cholangiocarcinoma. Annals of Surgical Oncology..

[41] Furukawa T, Higuchi R, Yamamoto M (2014). Clinical relevance of frozen diagnosis of ductal margins in surgery of bile duct cancer. Journal of Hepato-Biliary-Pancreatic Sciences..

